# Mechanical loading and fatigue-induced changes in triceps surae muscle-tendon unit: sex-specific responses and recovery dynamics

**DOI:** 10.3389/fphys.2026.1740634

**Published:** 2026-03-16

**Authors:** Saulė Salatkaitė-Urbonė, Robertas Lasevičius, Evi Wezenbeek, Danguolė Satkunskienė

**Affiliations:** 1 Institute of Sport Science and Innovations, Lithuanian Sports University, Kaunas, Lithuania; 2 Department of Health Promotion and Rehabilitation, Lithuanian Sports University, Kaunas, Lithuania; 3 Department of Rehabilitation Sciences and Physiotherapy, Ghent University, Ghent, Belgium

**Keywords:** achilles tendon, calf muscles, gender differences, mechanical properties, mechanical stress

## Abstract

This study investigates the effects of a repetitive plantar flexion fatigue protocol performed to volitional exhaustion on the contractility, structure, and passive mechanical properties of the triceps surae muscle-tendon unit (MTU) in young, physically active males and females. Twenty-two participants (10 females, 12 males) underwent a fatigue protocol consisting of repetitive bilateral standing calf raises, followed by assessments conducted pre-, post-, and 24 h after exercise. The study measured muscle contractility through peak torque, muscle and tendon structure using ultrasound echo intensity (EI), and mechanical properties via passive stiffness and hysteresis efficiency. Findings indicated a significant reduction in peak torque immediately after the fatigue protocol, alongside altered neuromuscular activation, with the gastrocnemius lateralis (GL) showing reduced EMG activity and the soleus (SOL) demonstrating compensatory recruitment. Muscle contractility exhibited partial recovery after 24 h, while tendon EI decreased progressively, suggesting prolonged structural changes. MTU passive stiffness increased immediately post-fatigue but decreased after 24 h, implying transient alterations in mechanical properties. Sex-related differences in MTU responses are present but limited, and recovery following fatigue occurs at different time scales across MTU tissues.

## Introduction

Mechanical load plays a crucial role in both muscle and tendon adaptation, as well as in the development of tendinopathy or muscle damage (Bohm et al., 2015). The interaction between mechanical signaling and biochemical changes within the tendon’s extracellular matrix or muscle connective tissues leads to adaptations in tissue morphology, structure, and material properties, such as strength, stiffness, and viscoelasticity, over time (Kjær et al., 2009; Wang, 2006). However, excessive loading, particularly without adequate recovery, can surpass the tendon’s or muscle’s capacity for connective tissue repair, resulting in fatigue-induced damage, which causes structural or functional changes in tissue that impair performance and heighten susceptibility to injury (Shepherd and Screen, 2013; Fang et al., 2024). Fatigue-induced damage is characterized as the progressive accumulation of microscopic structural harm. In cases of low fatigue, there are isolated deformations of kinked fibers (Fung et al., 2009). Moderate fatigue is characterized by increased kinking and a widening of the space between fibers. In instances of high fatigue, there is significant disruption of the matrix, misalignment of fibers, and further widening of the interface space (Fung et al., 2009). These matrix discontinuities arise from repeated mechanical loading over time and progressively compromise tissue strength and functional integrity (Fung et al., 2009). Evidence from various experimental studies indicates that matrix damage is an early sign of fatigue-related injury, occurring before noticeable changes in overall mechanical properties (Shepherd and Screen, 2013; Fang et al., 2024).

Investigating tendon fatigue *in vivo* is challenging, prompting the use of *in vitro*, animal, and computational models to better understand fatigue-induced structural changes (Shepherd and Screen, 2013; Glazebrook et al., 2008; Jafari et al., 2015; Szczesny et al., 2018; Zitnay et al., 2020; Herod and Veres, 2018). Animal studies have shown that high levels of repetitive loading can induce collagen molecular denaturation, increased cell density, collagen disorganization, and decreased fiber density, leading to changes in tendon structure such as increased fibril elongation and bowing, formation of kink bands, and reduced tissue stiffness (Glazebrook et al., 2008; Jafari et al., 2015; Szczesny et al., 2018; Zitnay et al., 2020; Herod and Veres, 2018). Similar patterns are observed in humans, although the magnitude and time course of tendon mechanical changes after fatiguing exercise appear highly dependent on the loading paradigm, contraction type, and assessment method (Shepherd and Screen, 2013; Shepherd et al., 2014; Joseph et al., 2014; Lepley et al., 2018; Pasquet et al., 2000; Kubo et al., 2001). For example, some studies report decreases in tendon stiffness following repeated hopping or isometric contractions (Joseph et al., 2014; Lepley et al., 2018; Lichtwark and Wilson, 2005), whereas others show minimal or transient changes (Pasquet et al., 2000; Kubo et al., 2001; Pro et al., 2001). These discrepancies are likely related to differences in measurement techniques, such as ultrasound elastography versus dynamometry-based approaches, as well as the timing of assessments relative to fatigue and recovery (Pasquet et al., 2000; Kubo et al., 2001).

Ultrasound-based techniques have become a standard approach to measure tendon mechanical properties *in vivo*, allowing non-invasive evaluation of tendon deformation and viscoelastic behavior by combining B-mode imaging with force measurements such as dynamometry. These methods provide insight into tendon mechanics and adaptations in humans under loading conditions (Seynnes et al., 2015).

Despite the well-established role of mechanical loading in the development of tendinopathy, precise changes in tendon structure and mechanical properties resulting from fatigue in humans remain incompletely understood, particularly under *in vivo* conditions (Piponnier et al., 2020). Fatigue likely affects tendon and muscle properties concurrently, altering tissue stiffness, activation patterns, and force transmission, yet the interplay between these changes is complex and context-dependent (Shepherd et al., 2014; Joseph et al., 2014; Lichtwark and Wilson, 2005). This highlights the importance of studying the muscle–tendon unit (MTU) as an integrated system rather than analyzing muscle or tendon in isolation, particularly when investigating injury risk and rehabilitation strategies. The triceps surae MTU, comprising the gastrocnemius lateralis, gastrocnemius medialis, soleus, and Achilles tendon (AT), contains multiple connective tissue components that contribute to passive tension, force transmission, and energy storage (Kubo et al., 2001; Finni, 2006). Understanding how these components respond to mechanical loading and fatigue is critical for preventing overload-related injuries and optimizing performance.

Sex-based differences in tendon mechanical responses have also been reported. Women may exhibit greater decreases in tendon stiffness following passive stretching or fatiguing hopping protocols, whereas men’s tendons are generally stiffer and less compliant, potentially influencing adaptation and injury risk (Dalmau-Pastor et al., 2014; Enoka and Duchateau, 2008; Burgess et al., 2009; Miller et al., 2007). However, responses can be task-dependent, and recovery dynamics vary across loading types and intensities (Enoka and Duchateau, 2008; Burgess et al., 2009; Astill et al., 2017). These findings underscore the complex and context-dependent nature of fatigue-induced changes in tendon mechanical properties in humans. Also, important that, fatigue-induced alterations of the muscle–tendon unit may differ between males and females due to sex-specific differences in tendon morphology, collagen turnover, and neuromuscular strategies (Miller et al., 2007; Hicks et al., 2001; Kjær et al., 2009). In the present study, fatigue refers specifically to an acute, exercise-induced state resulting from a single bout of repetitive mechanical loading, and does not reflect chronic fatigue, overuse, or long-term adaptive processes.

The aim of this study is to assess sex-specific changes in the contractility, structure, and mechanical properties of the MTU immediately following and 24 h after a repetitive plantar flexion fatigue protocol performed to volitional exhaustion in young, physically active males and females. We hypothesize that immediately following mechanical stress, MTU passive stiffness will decrease, including signs of mechanical fatigue, such as decreased tendon stiffness, echo intensity in muscle and tendon structure in both sexes. Furthermore, we expect that triceps surae MTU contractility will recover more quickly than tendon structure. However, due to sex differences in collagen synthesis and tendon adaptability, we anticipate that women will experience a slower recovery rate in tendon structure.

## Materials and methods

### Participants

Data were collected from a total of 22 healthy participants (12 males and 10 females) with the following characteristics: age 24.3 ± 4.2 years, height 1.77 ± 0.08 m, body mass 73.6 ± 11.1 kg, and BMI 23.5 ± 2.5 kg/m^2^ (mean ± SD). All participants were classified as physically active, defined as engaging in structured exercise programs for at least 1 year, with a frequency of at least three sessions per week. Regular exercise included a combination of resistance training and aerobic/endurance activities such as running, cycling, or gym-based workouts. Inclusion criteria required participants to have uninjured AT, no history of lower extremity injuries, and no health issues. Participants were instructed to refrain from vigorous physical activity for 24 h before the testing session and to maintain their regular diet. The study protocol was approved by the Kaunas Regional Biomedical Research Ethics Committee (No. BE-2-4, 2024-03-06) and adhered to the Declaration of Helsinki.

### Experimental design and measurements

This study employed an experimental design to assess various parameters related to the MTUt, including echo intensity of the AT, gastrocnemius lateralis (GL), and gastrocnemius medialis (GM); LON-sectional area (CSA) of the AT; passive stiffness (MTUSTF) and hysteresis of the MTU; and peak torque during maximal voluntary isometric contraction (MVIC). Participants were evaluated at three time points: before the fatigue protocol, immediately after, and 24 h post-protocol. All measurements were conducted on the right leg, which was the dominant limb in participants. The dominant leg was determined by asking the subject which leg they would use to kick a soccer ball. This standardization allowed for consistent data collection and reduced variability due to potential inter-limb asymmetries. The fatigue task was performed bilaterally to simulate natural movement and evenly distribute the load, while focusing measurements on a single leg simplified the protocol and improved measurement reliability.

The recruitment occurred between February 26 and May 31, 2024. Participants received an oral explanation of the study procedures, objectives, and potential risks, and provided written informed consent before enrollment. Anthropometric data, including height and body mass, were collected using a Tanita-305 body-fat analyzer (Tanita Corp.) and a KaWe PERSON-CHECK height stadiometer (Kirchner & Wilhelm GMBH + Co, KG). Participants then familiarized themselves with the testing equipment, and ultrasonography (US) was used to identify specific locations on the AT and the GM and GL muscles’ bellies for EI and CSA assessments.

Surface electromyography (EMG) electrodes were applied to the shaved, cleaned, and abraded skin over GL, GM, and SOL muscles following the European recommendations for surface electromyography (Hermens et al., 2025). Participants were positioned prone in the Biodex isometric device for MTUSTF and MVIC assessments. After baseline testing, the fatigue protocol was implemented, followed by immediate post-fatigue assessments, typically completed within 1–2 min. This brief transition time is consistent with prior studies, which report persistent reductions in neuromuscular function and mechanical properties for at least 2 min post-fatigue (Froyd et al., 2013; Obst et al., 2013). Participants were asked to avoid additional physical activity throughout the study. The 24-h post-protocol session repeated the same measurements, with delayed muscle soreness additionally recorded. The post-exercise assessments were conducted at a consistent time interval (24 ± 1 h) following the fatigue protocol for all participants to minimize the influence of time-dependent recovery processes.

### Achilles’ tendon and calf muscles ultrasound imaging

Participants lay prone with extended knees, lower legs on the treatment table, and feet hanging off the edge. Ultrasound was used to identify the distal muscle-tendon junction (MTJ) for the GM, GL and SOL and the osteotendinous junction (OTJ) at the calcaneus. These locations were marked on the skin (Barfod et al., 2015), and the distance between the OTJ and MTJ was measured using Rollfix tape. Three markers were placed on the skin to denote the 25% (proximal), 50% (middle), and 75% (distal) points along the free AT length between the soleus-Achilles MTJ and the Achilles-calcaneal OTJ (Kongsgaard et al., 2011).

**FIGURE 1:**
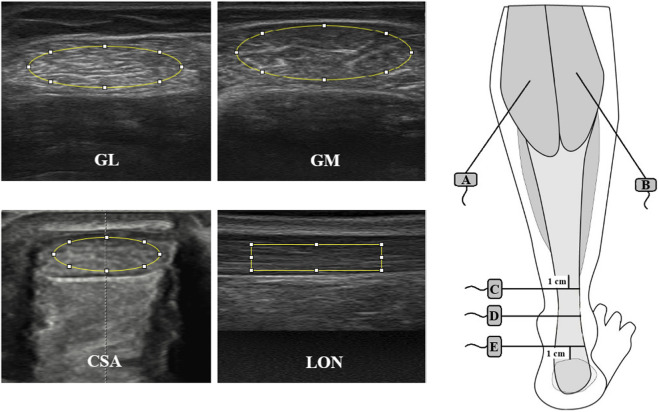
Ultrasound images showing measurement sites and regions of interest (ROI) for echointensity (EI) analysis of the Achilles tendon and gastrocnemius muscle. GL–gastrocnemius lateralis muscle cross-section area, GM–gastrocnemius medialis muscle cross-section area, CSA–cross-section area of Achilles tendon, LON–longitudinal area of Achilles tendon. (A) place of probe on gastrocnemius medialis, (B) place of probe on gastrocnemius lateralis, (C) place of probe on proximal part of Achilles tendon, (D) place of probe on medial part of Achilles tendon, (E) place of probe on distal part of Achilles tendon.

Grayscale B-mode images were captured using the Echoblaster 128 ultrasound system (UAB; Telemed, Vilnius, Lithuania) with a 10–15 MHz transducer. Longitudinal images of the AT and transverse images of GM, GL, and AT at proximal, middle, and distal locations were acquired (Crawford et al., 2023; Page et al., 2025). All ultrasound scan settings were standardized across all measurement sessions: muscle imaging (depth 6.0 cm, focus 1.5 cm, frequency 10 MHz, dynamic range 74 dB, gain to 87%), tendon imaging (depth 4.0 cm, focus 1.2 cm, frequency 10 MHz, dynamic range 74 dB, gain to 100%). Presets were saved before the first measurement session and locked for subsequent sessions. No post-processing filters or contrast adjustments were applied. Probe orientation was adjusted to maintain perpendicular insonation and minimize anisotropy, using fascicle alignment and avoidance of hypoechoic artifacts as visual guides. Ample gel was applied, particularly at the edges, forming “gel bridges” to prevent tissue compression (Lanferdini et al., 2019). Three images per site were captured by the same experienced sonographer and exported for analysis (Figure 1).

EI and CSA were quantified in ImageJ (NIH, Bethesda, United States). The mean EI was expressed in a grey-scale value (GSV) ranging from 0 (black) to 255 (white) (Lanferdini et al., 2019), with regions of interest (ROIs) placed at the muscle mid-belly or tendon mid-substance, avoiding artifacts. Oval ROIs (30 × 10 mm for muscle, 8 × 2.5 mm for transverse tendon) and rectangular ROIs (25 × 3 mm for longitudinal tendon) were used consistently across time points (Figure 1). EI reliability was assessed in one participant across 20 ultrasound sessions performed by the same operator. Images were analyzed twice, 24 h apart, in a double-blind manner. The average intraclass correlation coefficient (ICC) was 0.972 (95% CI 0.930–0.989), indicating excellent repeatability. The standard error of measurement (SEM) was 0.577 GSV, and the minimal detectable change at the 95% confidence level (MDC95) was 1.123 GSV. EI was selected because it has demonstrated biological and construct validity in previous work, showing associations with histological tissue composition, muscle function, and imaging markers of tissue quality (Pillen et al., 2009; Collinger et al., 2010).

The CSA was measured by defining the tendon borders inferior to the first hyperechoic region between the subcutaneous tissue and the deep fascia layer. The reliability analysis showed good test-retest reproducibility for AT CSA (ICC = 0.821, 95% CI 0.546 - 0.929). The SEM was 0.289 mm^2^, and the MDC95 was 0.566 mm^2^, indicating that only changes greater than approximately 0.6 mm^2^ should be interpreted as true physiological differences rather than measurement variability.

Midsection anterior-posterior thickness was measured perpendicular to the tendon, between the superficial and deep tendon borders, with the average of three images per location used for analysis.

### MTU passive stiffness and hysteresis

Participants lay prone without shoes on a Biodex System 4 isokinetic dynamometer (Biodex Medical Systems, Inc., Shipley, NY, United States), with the right foot secured to the footplate, aligning the rotational axis with the lateral malleolus. The dynamometer passively rotated the ankle from 10° plantarflexion to 30° dorsiflexion at 4°/s, returning to the starting position. Passive torques, ankle angle, and EMG from GL, GM, and SOL were synchronized using the Biopac system and recorded with AcqKnowledge 4.1.1 software (BIOPAC Systems, Inc.). Six repetitions were performed with 1-min rests, and the last three stretches were analyzed (Borges et al., 2021).

Before calculating the muscle-tendon unit passive stiffness (MTUSTF), muscle activity during the passive stretch was assessed. Measurements were accepted only when foot plantar-flexor activity was less than 5% of the maximal EMG value. MTUSTF was defined as the change in passive torque (PsT), adjusted for the mass of the foot, divided by the change in range of motion (ROM) (ΔPsT/ΔROM). The passive torque–angle relationship was fitted to a third-degree polynomial using the least squares method, and the R-squared value was used to assess the goodness of the fit (*R* ^2^ > 0.98) (Satkunskiene et al., 2020). Previous research (Finni, 2006) has shown that stiffness is not linear across the joint range. Therefore, stiffness was calculated as the slope of the passive torque–angle curve within its linear region, specifically for the ankle dorsiflexion ROM intervals of 20%–40%, 40%–60%, 60%–80%, and 80%–100% (Cesanelli et al., 1985). MTUSTF was normalized to body mass to account for individual size differences. For group-level analysis, individual torque–angle curves were averaged to generate mean passive torque–angle curves at each time point, providing a representative profile of passive mechanical behavior across participants (Kubo et al., 2010).

The integration method was used to calculate absorbed mechanical energy during the loading (stretching) phase and returned energy during the unloading (recovery) phase (Maganaris and Paul, 2000). Hysteresis efficiency was determined as the percentage of input energy recovered after one cycle of loading and unloading: Hysteresis efficiency = returned energy/absorbed energy × 100%. Higher hysteresis efficiency indicates greater energy recovery by the MTU, while lower efficiency reflects greater energy dissipation. MTUSTF, absorbed energy, dissipated energy, and hysteresis efficiency values from the three trials were averaged for subsequent analysis.

### Maximal voluntary isometric contractions

After measuring passive resistive torque, participants performed MVICs. A supervisor provided verbal encouragement and instructed participants to contract as forcefully as possible during each trial. Each participant completed three maximal contractions at a 90° ankle joint angle (neutral foot position), with each contraction lasting 3 s and a 2-min recovery period between attempts. The maximal voluntary isometric torque was defined as the peak torque achieved during these contractions. The peak torque value used for analysis, normalized by body mass, was taken from the best of the three attempts. Peak torque was calculated as the highest value obtained from a moving-average window during the plateau phase of the maximal voluntary isometric contraction. No formal threshold (e.g., 5% difference) was applied between trials, but all participants were encouraged to make maximal effort, and outliers were retested if deemed inconsistent.

### EMG signal processing

Surface EMG signals from the GL, GM, and SOL muscles were recorded using a Biopac MP100 data acquisition system (Biopac Systems Inc., Goleta, CA, United States) and AcqKnowledge software. EMG signals were sampled at 1000 Hz and synchronized with the dynamometer MVIC data. Bipolar Ag/AgCl surface electrodes were used with an inter-electrode distance of 20 mm (center-to-center). Prior to electrode placement, the skin was shaved, lightly abraded, and cleaned with alcohol to minimize skin impedance. Electrodes were placed over the muscle belly of each muscle and aligned parallel to the muscle fiber orientation according to the SENIAM (Surface EMG for Non-Invasive Assessment of Muscles) recommendations (Hermens et al., 2025). A reference electrode was positioned over a bony prominence. The EMG signals were filtered using a finite impulse response (FIR) band-pass filter with the Hamming window method. The filtered EMG amplitude was then transformed into a root mean square (RMS) value by applying a moving average filter with a 0.03-s window (Luca, 1997). The maximum EMG value was determined by calculating the mean RMS over 1 s centered around the peak torque during the MVICs. Immediately following the fatigue protocol EMG amplitudes were normalized to the maximum EMG obtained during the pre-fatigue MVICs for each muscle.

### Fatigue protocol

Before starting the fatigue protocol, participants performed a maximal bilateral standing calf raise against a wall. The highest point reached was marked at eye level, and participants were required to touch the ground with their heels to ensure a full range of motion. This served as a reference for their distance during the fatigue protocol. The protocol consisted of metronome-paced bilateral calf raises at 70 per minute, each set lasting 60 s with a 120-s rest sitting on a chair. The total number of sets was recorded, and individual effort after each set was monitored via the Borg CR10 scale (Borg, 1982). The fatigue protocol was stopped once participants reached maximum effort (Borg CR10 scale ≥9), or they were unable to attain the highest position. Maximal effort and exhaustion were confirmed using a combination of objective and subjective criteria. Exhaustion was defined as the inability to reach the individualized reference height for two consecutive repetitions, despite strong verbal encouragement, and/or the inability to maintain the prescribed cadence. In addition, perceived exertion was assessed immediately after each set using the Borg CR10 scale, with maximal effort confirmed when participants reported values indicative of near-maximal or maximal exertion (≥9). Comparable levels of maximal effort across participants were ensured by using individualized performance-based termination criteria rather than a fixed workload or number of repetitions. Standardizing workload across participants was avoided to prevent under- or overloading, which could confound tendon EI measurements.

### Delayed muscle soreness evaluation

Twenty-four hours after completing the fatigue protocol, participants were asked to rate their calf soreness on a 10-point rating scale, where 0 indicated “no pain” and 10 represented “extreme pain” (Lau et al., 2013). Acute muscle soreness was not assessed in this study because it does not indicate structural damage to muscles or tendons. Instead, our focus was on delayed onset muscle soreness, which is more closely related to the timing and mechanism of tissue disruption following eccentric loading (Pro et al., 2001).

### Statistical analysis

IBM SPSS Statistics version 21.0 (IBM, Armonk, United States) was used for all data analysis. Descriptive statistics (mean ± SD) were calculated for each variable, and the Shapiro-Wilk test was applied to assess the normality of the sample distribution. All variables were found to be normally distributed (p > 0.05). Independent t-tests were carried out to determine if there were significant differences in the mean values of all tested variables between the groups (female vs. male). The ES was determined based on Cohen’s guidelines, with the standardized mean difference (d) and pooled standard deviation interpreted as trivial, <0.20; small, 0.20–0.59; moderate, 0.60–1.19; large, 1.20–1.99; and very large, ≥2.00 (Fritz et al., 2011). Dependent variables were analyzed using Mixed ANOVA for repeated measures, with Bonferroni correction applied to pairwise comparisons of main effects. Time (days), group (female vs. male), and interaction effects were also evaluated. The effect size (ES) was quantified using partial eta-squared (η_p_^2^) for repeated measures, with magnitude classifications as minor (<0.02), moderate (<0.13), and large (<0.26) (Bakeman, 2005). The effect size for pairwise comparisons was determined according to Cohen’s guidelines for paired samples, where the mean difference between paired measurements was divided by the standard deviation of the differences. Effect sizes were classified as trivial (d < 0.20), small (0.20–0.49), medium (0.50–0.79), or large (d ≥ 0.80) (Luca, 1997). Pearson correlation analysis was used to assess the relationship between morphological and mechanical properties of the triceps surae muscle-tendon unit. The Pearson correlation coefficient was interpreted as follows: very strong (r = 0.9–1.0), strong (r = 0.7–0.9), moderate (r = 0.5–0.7), weak (r = 0.3–0.5), and very weak (r = 0–0.3). A significance level of 0.05 was set as the threshold for statistical significance.

## Results

Males exhibited significantly greater height and mass (p < 0.001), as well as greater AT-GM and AT-GL measurements (p < 0.05) compared to females (Table 1). However, no significant differences were observed between males and females in terms of age, BMI, AT-SOL, the number of series completed during the fatigue protocol, or delayed muscle soreness 24 h post-protocol.

**TABLE 1:** Characteristics of the participants by sex (mean (SD)).

Variables	Female (n = 10)	Male (n = 12)	*p*	Cohen’s d
Age (years)	25 (4.74)	23.67 (3.87)	0.485	0.315
Height (m)	1.70 (0.05)	1.82 (0.06)	<0.001	1.419
Mass (kg)	65.04 (5.35)	80.75 (9.55)	<0.001	1.410
BMI (kg/m^2^)	22.43 (1.75)	24.43 (2.65)	0.055	0.815
AT-GM (cm)	17.26 ± 1.99	20.05 (3.04)	0.022	0.954
AT-GL (cm)	20.39 (1.72)	22.3 (1.36)	0.011	1.071
AT-SOL (cm)	5.53 (0.84)	5.75 (1.29)	0.647	0.203
Total sets of calf raise	13.5 (4.84)	16.33 (6.33)	0.248	0.493
Total calf raises	945 (339)	1143 (443)	0.260	0.493
Delayed muscle soreness after 24 h	5.7 (2.41)	4.58 (1.62)	0.230	0.546

SD, standart deviation; BMI, body mass index; GL, gastrocnemius lateralis; GM, gastrocnemius medialis; SOL, soleus; AT, achilles tendon.

### MTU echointencity

A repeated-measures ANOVA revealed a significant time effect on EI at the distal (p < 0.001; F = 29.155; ƞ_p_^2^ = 0.588), middle (p < 0.001; F = 34.682; ƞ_p_^2^ = 0.857) and proximal (p < 0.001; F = 27.314; ƞ_p_^2^ = 0.580), portions of the AT (Table 2). No significant sex × time interactions were detected for EI in any AT region. Immediately following the fatigue protocol, EI showed a tendency to decrease in both males and females across all AT portions, with a significant reduction observed at the distal AT in males (p = 0.027; d = 1.023). At 24 h post-fatigue, EI was significantly reduced in both sexes across all AT regions (p < 0.001; d > 1.2). Although no sex × time interaction was present, females generally exhibited higher EI values. Specifically, significantly greater EI in females was observed at the distal AT immediately after the fatigue protocol (p = 0.030; d = 1.018), as well as in the longitudinal AT under pre-fatigue (p = 0.016; d = 1.092), post-fatigue, and 24-h post-fatigue conditions (p < 0.001; d = 1.5).

**TABLE 2:** Mean (SD) of echo intensity (EI) in the Gastrocnemius lateralis (GL), Gastrocnemius medialis (GM), and Achilles tendon (AT) in males and females following a fatigue protocol (pre, post, and 24-h post).

Region	Male			Female		
	PRE	POST	POST 24 h	PRE	POST	POST 24 h
AT distal	57.66 (4.3)	53.85 (5.43)^a^	50.83 (4.97)^b^	60.84 (4.29)	59.16 (5.14)^d^	53.19 (4.43)^bc^
AT middle	55.72 (5.23)	52.79 (6.76)	48.64 (6.31)^b^	59.16 (5.91)	58.24 (6.7)	52.82 (5.24)^bc^
AT prox	52.89 (6.72)	49.26 (6.13)	46.43 (4.61)^b^	57.99 (5.16)	54.59 (7.52)	50.59 (7.47)^b^
AT long	42.01 (7.61)	36.50 (4.87)^a^	34.06 (4.43)^b^	50.86 (8.06)^d^	45.79 (6.25)^d^	43.26 (6.70)^bd^
GM	51.37 (11.55)	52.25 (8.00)	50.69 (6.05)	56.65 (10.52)	55.01 (6.77)	52.89 (7.07)
GL	55.84 (11.41)	56.55 (12.23)	55.22 (7.67)	67.71 (13.61)^d^	65.07 (12.93)	63.56 (9.45)^d^

SD = standart deviation; AT prox = proximal Achilles tendon; AT long = longitudinal Achilles tendon view. *p* < 0.05 for PRE vs. POST (a), PRE vs. POST-24 h (b), and POST vs. POST-24 h (c). Sex differences at the same time point: d *p* < 0.05.

In contrast, no significant main effects of time were found for EI in the GL or GM, and no sex × time interactions were observed. Nevertheless, females tended to exhibit higher muscle echointencity, with significantly greater EI in the GL muscle under pre-fatigue and 24-h post-fatigue conditions (p < 0.05; 1.00 < d < 1.20).

### AT cross-section area

A significant main effect of time was observed for the cross-sectional area (CSA) of the middle portion of the Achilles tendon (AT) (F = 4.028; p = 0.025; ηp^2^ = 0.168), accompanied by a near-significant sex × time interaction (F = 3.166; p = 0.053; ηp^2^ = 0.137). In females, CSA in the middle AT region was significantly lower immediately after the fatigue protocol compared with pre-fatigue values (p = 0.015; d = 0.944) (Table 3). Across all time points, females exhibited significantly smaller CSA in the distal (p < 0.01; 1.216 < d < 1.375) and proximal AT regions (p < 0.01; 1.092 < d < 1.426), as well as in the middle AT region immediately post-fatigue (p < 0.05; d = 0.962), compared with males.

**TABLE 3:** Mean (SD) of cross-sectional area (mm^2^) of the Achilles tendon (AT) at the distal, middle, and proximal portions, as well as the anterior-posterior thickness (mm) of the middle section of the AT in males and females following a fatigue protocol (pre, post, and 24-h post).

Region	Male			Female		
	PRE	POST	POST 24 h	PRE	POST	POST 24 h
AT distal	67.05 (9.52)	64.61 (7.82)	64.13 (6.22)	50.28 (8.11)^d^	49.65 (8.41)^d^	53.17 (8.41)^d^
AT middle	55.05 (8.19)	54.49 (8.96)	55.44 (8.99)	50.27 (10.08)	45.04 (8.53)^ad^	48.26 (10.50)
AT prox	57.04 (8.50)	55.33 (6.51)	56.23 (7.93)	46.96 (6.71)^d^	43.61 (4.77)^d^	46.59 (6.96)^d^
AT thickness	5.01 (0.35)	4.93 (0.48)	5.21 (0.43)	4.59 (0.41)^d^	4.47 (0.37)^d^	4.71 (0.42)^d^

SD, standart deviation; AT, prox = proximal Achilles tendon; a PRE, vs. POST, d females vs. males at the same time point (*p <* 0.05).

A significant main effect of time was also identified for the anterior–posterior thickness of the middle AT region (F = 6.046; p = 0.005; ηp^2^ = 0.232), with no significant sex × time interaction. Nevertheless, females consistently demonstrated significantly lower AT thickness than males across all time points (p < 0.05; 0.949 < d < 1.020).

### Plantar flexors’ contractile properties

**FIGURE 2:**
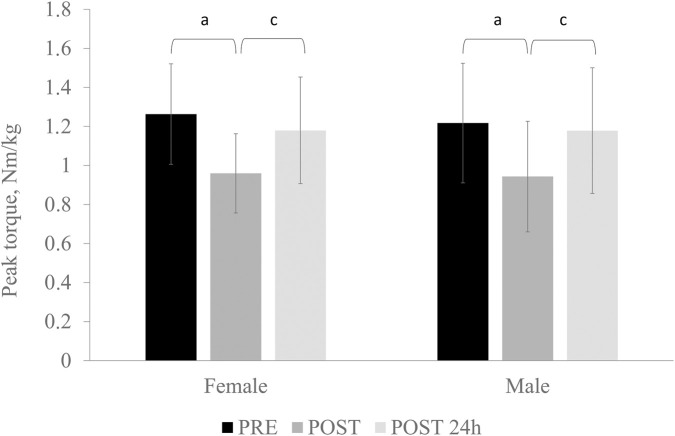
Normalized peak plantar-flexion torque in males and females following a fatigue protocol (pre, post, and 24-h post). Vertical error bars represent the standard deviation. *p* < 0.05 for PRE vs. POST (a) and POST vs. POST-24 h (c).

A significant main effect of time was observed for normalized peak torque (p < 0.001; F = 34.835; η^2^ = 0.635), with no significant sex × time interaction. Normalized peak torque did not differ significantly between females and males at any time point. Immediately following the fatigue protocol, normalized peak torque decreased significantly in both sexes (p < 0.001; d = 1.467). After 24 h, normalized peak torque increased significantly in both females (p = 0.003; d = 1.625) and males (p < 0.001; d = 1.103) compared with the post-fatigue condition. No significant differences were observed between pre-fatigue and 24-h post-fatigue measurements (Figure 2).

Immediately following the fatigue protocol, nEMG of the GL decreased significantly in both females (p < 0.001, d = 2.75) and males (p < 0.001, d = 1.58). Post-fatigue GL nEMG was significantly lower in females (62.6% ± 13.6%) than in males (76.2% ± 15.0%) (p = 0.039, d = 0.87). SOL nEMG increased immediately after fatigue in both sexes, reaching statistical significance in males (119.3% ± 34.9%; p = 0.048, d = 0.55) and showing a strong trend in females (116.8% ± 27.1%). In contrast, GM nEMG showed a pronounced tendency to decrease after fatigue in females (88.4% ± 13.6%; p = 0.084, d = 0.86), whereas no meaningful change was observed in males (90.8% ± 24.3%). No significant sex differences were detected for SOL or GM nEMG responses.

### MTU passive stiffness

**FIGURE 3:**
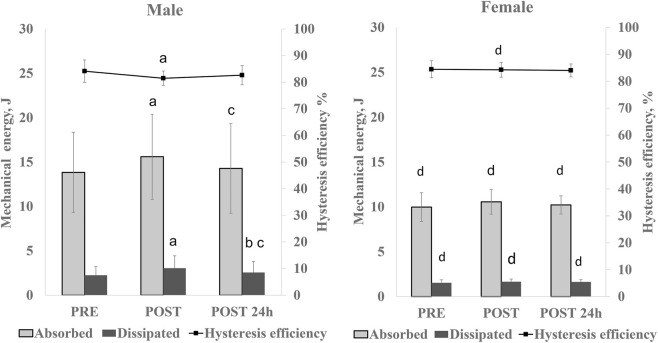
Absorbed energy during loading, dissipated energy during unloading and hysteresis efficiency for females and males at pre-, post-, and 24-h post-fatigue protocol time points. Vertical error bars represent the standard deviation. *p* < 0.05 for PRE vs. POST (a), PRE vs. POST-24 h (b), and POST vs. POST-24 h (c). Sex differences at the same time point: d *p* < 0.05.

A statistically significant main effect of time on MTUSTF (Table 4) was observed across all ranges of motion (ROM): 20%–40% (*F* = 14.033; *p* < 0.001; η^2^ = 0.412), 40%–60% (*F* = 12.933; *p* < 0.001; η^2^ = 0.393), 60%–80% (*F* = 5.007; *p* = 0.011; η^2^ = 0.200), and 80%–100% (*F* = 3.406; *p* = 0.043; η^2^ = 0.146). No significant sex × time interactions were detected. Immediately after the fatigue protocol, MTUSTF increased significantly in males at 20%–40% ROM (*p* < 0.001; *d* = 1.161), whereas the increase in females did not reach statistical significance (*p* = 0.086; d = 0.841) (Figure 3). At 40%–60% ROM, MTUSTF increased significantly in males (*p* < 0.001; *d* = 1.507), while females showed a strong trend toward an increase (*p* = 0.065; d = 0.808). At 60%–80% ROM, a significant increase in MTUSTF was observed only in males (*p* = 0.001; *d* = 1.290). No significant changes were detected at 80%–100% ROM in either sex immediately after fatigue. After 24 h, MTUSTF at 20%–40% and 40%–60% ROM decreased significantly in males (*p* < 0.001; d > 1.508), and no significant differences in MTUSTF were observed relative to pre-fatigue values in either sex across all ROMs. Across time points, females exhibited significantly lower absolute MTUSTF values than males at all ROMs (p < 0.05; 0.852 < d < 1.177), except at 80%–100% ROM under pre-fatigue and 24-h post-fatigue conditions, and at 60%–80% ROM at 24 h post-fatigue. However, when MTUSTF was normalized to body mass, no significant differences were observed between females and males.

**TABLE 4:** Mean (SD) of the absolute (MTU) and normalized (NBM) Triceps surae muscle-tendon unit passive stiffness in males and females following a fatigue protocol (pre, post, and 24-h post).

Force interval	Male			Female		
	PRE	POST	POST 24 h	PRE	POST	POST 24 h
MTU 20%-40% Nm/°	1.07 (0.30)	1.21 (0.33)^a^	1.06 (0.34)^c^	0.78 (0.08)^d^	0.85 (0.09)^d^	0.78 (0.07)^d^
NBM x 10^-2^ Nm⋅kg^-1^	1.31 (0.23)	1.48 (0.27)^a^	1.30 (0.30)^c^	1.21 (0.12)	1.30 (0.12)	1.20 (0.14)
MTU 40%-60%. Nm/°	1.29 (0.38)	1.46 (0.43)^a^	1.32 (0.45)^c^	0.96 (0.13)^d^	1.02 (0.14)^d^	0.99 (0.09)^d^
NBM x 10^-2^ Nm⋅kg^-1^	1.58 (0.31)	1.78 (0.35)^a^	1.61 (0.38)^c^	1.47 (0.16)	1.58 (0.16)	1.52 (0.16)
MTU 60%-80%. Nm/°	1.64 (0.56)	1.77 (0.56)^a^	1.71 (0.60)	1.23 (0.26)^d^	1.28 (0.22)^d^	1.30 (0.22)
NBM x 10^-2^ Nm⋅kg^-1^	1.99 (0.46)	2.16 (0.46)^a^	2.08 (0.51)	1.88 (0.32)	1.96 (0.24)	2.00 (0.31)
MTU 80%-100% Nm/°	2.11 (0.80)	2.16 (0.72)	2.22 (0.81)	1.58 (0.47)	1.60 (0.34)^d^	1.71 (0.42)
NBM x 10^-2^ Nm⋅kg^-1^	2.56 (0.68)	2.63 (0.60)	2.70 (0.70)	2.41 (0.59)	2.44 (0.41)	2.64 (0.58)

SD, standart deviation; *p* < 0.05 for PRE, vs. POST (a) and POST, vs. POST-24 h (c). Sex differences at the same time point: d *p* < 0.05.

### MTU hysteresis

The analysis revealed a significant main effect of time on both input energy during loading (absorbed energy) and energy dissipated during unloading (p < 0.001; F = 11.394; η^2^ = 0.363 and p < 0.001; F = 11.054; η^2^ = 0.356, respectively). The effect of time on hysteresis efficiency showed a strong trend but did not reach statistical significance (p = 0.065; F = 2.928; η^2^ = 0.128). Significant sex × time interactions were observed for absorbed energy (p = 0.046; F = 3.329; η^2^ = 0.143) and dissipated energy (p = 0.005; F = 6.119; η^2^ = 0.234), whereas no sex × time interaction was detected for hysteresis efficiency. Immediately following the fatigue protocol, absorbed and dissipated energy increased significantly in males (p < 0.001; d = 1.394), accompanied by a significant decrease in hysteresis efficiency (p = 0.017; d = 0.881). No significant changes were observed in females at this time point. At 24 h post-fatigue, absorbed (p < 0.001; d = 1.616) and dissipated energy (p = 0.005; d = 0.956) in males decreased significantly compared with post-fatigue values; however, dissipated energy remained significantly elevated relative to baseline (p = 0.043; d = 0.768). No significant changes were detected in females across time.

Across time points, absorbed and dissipated energy were significantly lower in females compared with males under pre-fatigue and 24-h post-fatigue conditions (p < 0.05; 0.835 < d < 0.976), with the greatest sex differences observed immediately after the fatigue protocol (p < 0.01; 1.102 < d < 1.139). Immediately following the fatigue protocol hysteresis efficiency was significantly greater in females (p = 0.028; d = 0.921).

### Correlation analysis

Correlation analyses were performed using pooled data from females and males to ensure sufficient statistical power and to examine overall structure–function relationships rather than sex-specific associations. A weak positive correlation was observed between hysteresis efficiency and EI in the middle portion of the AT under both the pre-fatigue condition and 24 h post-fatigue (p < 0.05) (Table 5). Additionally, a moderate positive correlation was found between hysteresis efficiency and EI in the distal and middle portions of the AT immediately following the fatigue protocol (p < 0.01). A weak positive correlation was also observed between hysteresis efficiency and EI in the proximal portion of the AT, as well as with EI measured via longitudinal ultrasonography of the AT (p < 0.05).

**TABLE 5:** Pearson correlation (r) with 95% confidence intervals between hysteresis efficiency and Achilles tendon (AT) echo intensity (EI) at distal, middle and proximal portions for pooled male and female data at pre-, post-, and 24-h post-fatigue protocol time points.

Condition	Variables	Hysteresis efficiency r	*p*	95% confidence intervals	
				Lower	Upper
PRE	EI AT middle	0.496	0.019	0.083	0.754
POST	EI AT distal	0.612	0.002	0.243	0.817
	EI AT middle	0.552	0.008	0.157	0.785
	EI AT prox	0.436	0.042	0.007	0.72
	EI AT long	0.479	0.024	0.061	0.744
POST 24 h	EI AT middle	0.455	0.034	0.03	0.73

EI, echo intensity; AT, achilles tendon; prox = proximal AT; long = longitudinal AT, view.

## Discussion

The primary aim of this study was to examine the sex-specific changes in MTU contractility, structure, and mechanical properties immediately following, and 24 h after, a repetitive plantar flexion fatigue protocol performed until volitional exhaustion in young, physically active adults. The main finding was that both males and females exhibited similar levels of neuromuscular fatigue and recovery at the contractile level. However, sex-specific differences were observed in the structural and mechanical responses of the MTU following the fatigue protocol and during the subsequent recovery period. These results suggest that the adaptations to acute fatigue differ by sex, primarily manifesting at the tendon and MTU mechanical level rather than through variations in muscle strength loss or recovery.

### Sex-specific structural characteristics of the achilles tendon

The present study shows that females consistently had smaller CSA and anterior–posterior AT thicknesses across most regions, along with higher EI values in both the AT and plantar flexor muscles. Fatigue-induced decrease in EI in the tendon occurred similarly in both males and females, as there were no significant interactions between sex and time. This finding indicates that mechanical loading modifies the acoustic properties of the tendon in both sexes, regardless of different structural baselines, and is consistent with our previous study (Salatkaite et al., 2025).

Changes in ultrasound EI in muscles and tendons are typically associated with changes in non-contractile tissue content, intratendinous fluid distribution, edema and inflammation (Matta et al., 2018; W et al., 2020). Previous work has shown that the change in grayscale intensity in muscles was most evident from 48 to 96 h after loading (Cabral et al., 2021), which likely explains the lack of detectable changes at the 24-h time point in our study. In contrast, AT EI decreased across distal, middle, and proximal regions after 24 h. Since the CSA of AT did not increase at this time point, reductions in AT EI may reflect subtle changes in collagen organization or fiber alignment following loading rather than edema (Roesch et al., 2025; Syha et al., 2014; Garcia et al., 2003; Ishigaki et al., 2016). However, it's important to note that echointensity remains an indirect surrogate of tendon structure and should be interpreted with caution.

### Contractile fatigue and neuromuscular coordination

Both sexes experienced significant exercise-induced muscle fatigue, indicated by an approximately 23% reduction in peak torque immediately after the exercise protocol (Finsterer, 2012). This was accompanied by a decrease in GL EMG activity by 30% ± 16% and an increase in SOL EMG activity by 18% ± 31% during maximal voluntary contraction. This pattern likely reflects the reduced contractile capacity of the more fatigable GL and the compensatory recruitment of the fatigue-resistant SOL, aligning with known fiber-type distributions and previous studies (Enoka and Duchateau, 2008; Edgerton et al., 1975; Gollnick et al., 1974; Kallenberg et al., 2007).

While most neuromuscular responses were similar between sexes, females showed a greater reduction in GL EMG activity immediately after fatigue, which may reflect sex-specific differences in neuromuscular activation strategies or modulation of neural drive to the gastrocnemius (Hunter, 2014). These differences in activation strategy may impact tendon loading patterns, especially considering the complex subtendon architecture of the AT. The AT consists of three distinct subtendons that twist as they descend towards the calcaneal insertion, with the largest subtendon originating from the GL and experiencing the greatest degree of torsion (P et al., 2017; Winnicki et al., 2020). Therefore, fatigue-induced changes in muscle fibers recruitment could redistribute tensile forces across different tendon regions, potentially increasing local strain patterns or the risk of injury.

### Sex differences in MTU passive mechanics

In contrast to the generally similar contractile responses observed, significant differences between sexes emerged in terms of MTU passive stiffness and energy-related outcomes. Males demonstrated clear fatigue-induced increases in passive MTU stiffness, particularly at low-to-moderate ranges of motion, whereas females showed only non-significant trends toward increased stiffness. Similar transient increases in stiffness following fatiguing or eccentric exercise have been attributed to short-term changes in contractile elements, connective tissue behavior, and intramuscular fluid distribution (Howell et al., 1993; Joumaa et al., 2008; Labeit et al., 2003; Whitehead et al., 2001). The present findings suggest that males respond to acute fatigue with a mechanically stiffer MTU, potentially enhancing force transmission but increasing mechanical loading on the tendon.

MTU stiffness changes were accompanied by distinct energetic responses. Following fatigue, males exhibited significant increases in absorbed and dissipated energy, along with a reduction in hysteresis efficiency, indicating greater energy loss during loading–unloading cycles. The increased dissipated energy in males 24 h after fatigue, despite normalization of stiffness and peak torque, indicates delayed recovery of tendon viscoelastic behavior. In contrast, females showed remarkable stability in absorbed energy, dissipated energy, and hysteresis efficiency across time. The dissociation between contractile recovery and tendon energetics highlights the importance of assessing MTU mechanical properties independently of muscle strength.

### Tendon structure - function relationships

Correlation analyses further establish a connection between tendon structure and mechanical behavior of MTU. Weak positive correlations between hysteresis efficiency and EI in the middle portion of the Achilles tendon were observed before loading and after 24 h, with moderate correlations immediately post-fatigue. These associations suggest that tendon microstructural integrity influences viscoelastic behavior during cyclic loading (Thorpe et al., 2013). Accordingly, the observed positive relationships indicate that tendons with higher EI tend to exhibit greater hysteresis efficiency, suggesting reduced energy loss and more effective elastic energy return within the muscle–tendon unit (McNeill, 2002). The stronger correlations observed immediately post-fatigue may reflect an acute amplification of the coupling between tendon structure and function following repetitive mechanical loading (Magnusson et al., 2010).

### Recovery and fatigability considerations

Mechanical load and delayed muscle soreness did not significantly differ between females and males. Sex differences in fatigability are known to diminish at high contraction intensities (Hicks et al., 2001; Maughan et al., 1986), which may explain the broadly similar fatigue and recovery profiles observed at the contractile level in our study. Both sexes demonstrated partial recovery of torque at 24 h, accompanied by moderate delayed-onset muscle soreness consistent with previous reports following high-volume loading (O’Malley et al., 2024).

## Limitations

Several limitations should be acknowledged. First, the 24-h recovery window may be insufficient to capture the full temporal profile of muscle and tendon recovery; future studies should extend follow-up to 72–96 h and include direct measures of tendon stiffness. Second, echointensity is influenced by hydration, edema, and biological variability, limiting its specificity as a marker of tendon microstructure. Incorporation of texture-based analyses, such as peak spatial frequency, may provide more robust structural insights. Third, the absence of objective markers of baseline MTU micro-damage (e.g., creatine kinase) introduces potential variability in pre-fatigue conditions. Finally, limb dominance was determined by self-report, which may not accurately reflect functional dominance of the ankle plantar flexors.

## Conclusion

In summary, males and females exhibited comparable neuromuscular fatigue and recovery following exhaustive plantar flexion exercise, yet demonstrated distinct structural and mechanical MTU responses. Males showed greater fatigue-induced stiffness and energy dissipation, whereas females maintained more stable viscoelastic behavior and elastic efficiency. Although muscle contractility demonstrated partial recovery within 24 h in both sexes, recovery dynamics differed across MTU components, suggesting distinct adaptation time scales between muscle and tendon tissues. Passive MTU stiffness and hysteresis largely normalized within 24 h, whereas tendon echo intensity remained reduced, indicating delayed structural recovery of the Achilles tendon. These sex-specific mechanical strategies may have important implications for tendon loading tolerance, injury risk, and the development of sex-informed training and rehabilitation protocols.

## Data Availability

The raw data supporting the conclusions of this article will be made available by the authors, without undue reservation.
